# Improvement of three-dimensional motion sickness using a virtual reality simulator for robot-assisted surgery in undergraduate medical students: A prospective observational study

**DOI:** 10.1186/s12909-021-02872-9

**Published:** 2021-09-21

**Authors:** Ryo Takata, Mitsugu Kanehira, Yoichiro Kato, Tomohiko Matsuura, Renpei Kato, Shigekatsu Maekawa, Wataru Obara

**Affiliations:** Department of Urology, Iwate Medical University, 1-1-1 Idaidori, Shiwa-gun, Iwate Prefecture 028-3694 Yahaba-cho, Japan

**Keywords:** virtual reality, motion sickness, three-dimensional, robotic-assisted surgery, da Vinci robot

## Abstract

**Background:**

A virtual reality (VR) simulator is utilized as an inexpensive tool for gaining basic technical competence in robotic-assisted surgery (RAS). We evaluated operator 3D motion sickness while using a VR simulator and assessed whether it can be reduced by repeating the training.

**Methods:**

This prospective observational study was conducted at the Department of Urology, Iwate Medical University, a tertiary training hospital in an urban setting. A total of 30 undergraduate medical students participated in the study. We compared whether the VR simulator improved the students’ skills in operating the da Vinci robot. Fifteen students underwent training with a VR simulator for 4 h a day for 5 days. Then, motion sickness was determined using the Visual Analog Scale and Simulator Sickness Questionnaire (SSQ) before and after the training.

**Results:**

Manipulation time significantly improved after training compared to before training (293.9 ± 72.4 versus 143.6 ± 18.4 s; *p* < 0.001). Although motion sickness worsened after each training session, it gradually improved with continuous practice with the VR simulator. SSQ subscores showed that the VR simulator induced nausea, disorientation, and oculomotor strain, and oculomotor strain was significantly improved with repeated training.

**Conclusions:**

In undergraduate students, practice with the VR simulator improved RAS skills and operator 3D motion sickness caused by 3D manipulation of the da Vinci robot.

**Supplementary Information:**

The online version contains supplementary material available at 10.1186/s12909-021-02872-9.

## Background

Recently, the number of robot-assisted surgeries (RASs) has been increasing yearly, with the number of RASs using the da Vinci surgical system (Intuitive Surgical, Sunnyvale, CA, USA) reaching 1,200,000 in 2019 [1]. However, the increase in RASs augmented the number of iatrogenic injuries caused by improper use of the da Vinci robot [2]. Since a lack of experience has caused 53 % of surgical errors [3], strict training before performing RAS is required to evaluate and improve the surgeon’s competence [4]. In Japan, the Japanese Urological Association has formulated an educational program for performing RAS, which requires more than 20 h of training on the da Vinci robot [5]. However, several hospitals cannot afford to purchase a da Vinci robot for training purposes; hence, a virtual reality (VR) simulator, an inexpensive tool for gaining basic technical competence in RAS, was developed [6]. Currently, there are six VR simulator types for RAS worldwide [7], with the da Vinci Skills Simulator (dVSS; Intuitive Surgical) as the most preferred because of its ergonomics and usability [8].

Since there is a lack of high-level evidence on whether VR simulators can guide a surgeon to the proficiency level necessary to perform RAS, various studies have investigated the efficacy and reproducibility of VR simulators [9–11]. However, they have not considered the operator’s fatigue caused by gazing at three-dimensional (3D) images in VR. Recent dramatic advances in video presentation technology have made it possible to construct 3D models in the medical field. Moreover, it can cause symptoms of physical fatigue, such as dizziness, headache, and nausea, i.e., visually induced motion sickness, and these symptoms are problematic [12].

Therefore, we evaluated the adverse effect of 3D motion sickness using a VR simulator and investigated whether repeated training with the VR simulator would improve 3D motion sickness in undergraduate medical students as part of the medical educational curriculum.

## Methods

### Research subjects

To evaluate the adverse effect of 3D motion sickness using a VR simulator and investigate whether repeated training with the VR simulator would improve 3D motion sickness in undergraduate medical students as part of medical educational curriculum, we conducted a prospective observational study at our institute in accordance with the principles of the Declaration of Helsinki. Participants were informed of the aims of this exploration, their right to refuse participation, and their right to withdraw from the study at any time. All participants provided written informed consent prior to study participation and data publication.　 Ethical approval　was waived by the institutional ethics committee of the Iwate Medical University School of Medicine.

Thirty students who were third- and fourth-year medical school students at the university hospital in an urban city were recruited by an open unpaid call (Table 1). Exclusion criteria were previous experience in clinical practice or surgical participation or prior experience with a VR simulator. In this study, the sample size was limited due to the coronavirus disease 2019 (COVID-19) pandemic. After obtaining written consent, the students followed the first instruction in the da Vinci robot’s essential operation. Then, 30 medical students were randomized into the trained or non-trained group. Fifteen students were enrolled in the trained group.

**Table 1:** Changes in the ability to manipulate the da Vinci robot through simulator training

	Total (*n *= 30)	Trained group (*n* = 15)	Non-trained group (*n* = 15)	*p*
Age, mean ± SD (years)	22.6 ± 1.56	22.4 ± 1.35	22.8 ± 1.76	**0.424**
Sex (Male / Female)	21 / 9	9 / 6	12 / 3	**0.223**
VR game experience (Yes / No)	24 / 6	12 / 3	12 / 3	**1.000**
Time to complete skill drill				
Pre-training (sec ± SD)	-	293.9 ± 72.4	350.5 ± 125.3	**0.142**
Post-training (sec ± SD)	-	143.6 ± 18.4	257.1 ± 70.0	**< 0.001**
***p***		**< 0.001**	**0.003**	
SD, standard deviation				

### Training with the da Vinci robot

**Fig. 1:**
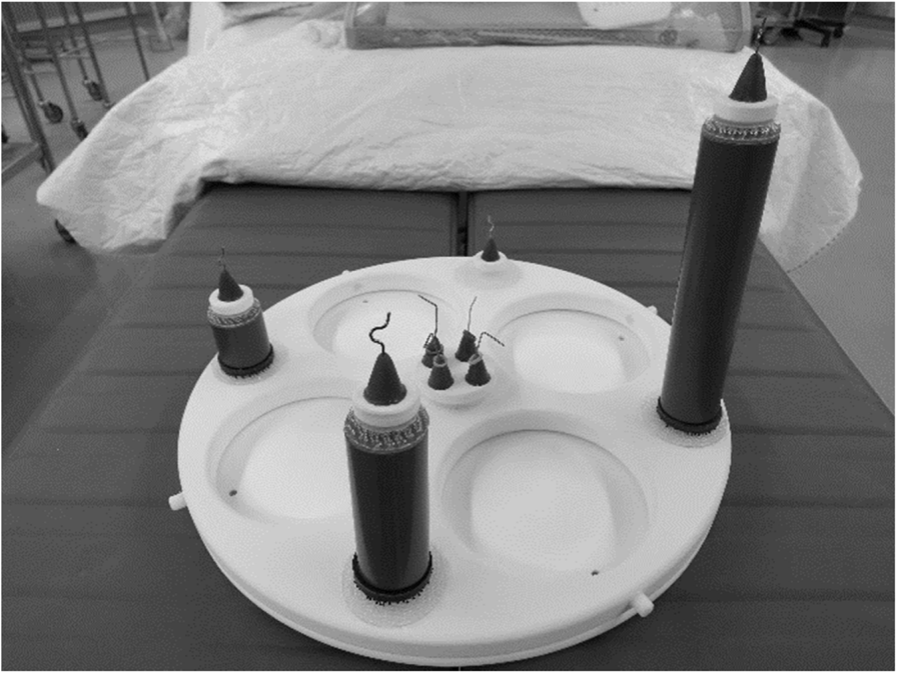
Manipulation model. There are eight columns on the round table. Four columns are located at the center, and the other columns are placed on the perimeter of the round table. The figure shows “skill drill” training wherein a rubber band placed on the center projection is moved to the outer circumference and then back to the center column using the da Vinci instruments

We used the manipulation model (Intuitive Surgical) with the da Vinci robot to perform the “skill drill” training. This model consists of a round table with four short columns at the center and four columns of different heights arranged evenly at 90° around the periphery of the table (Fig. 1). At the start of training, the trainee picked up the rubber bands placed on each central column with one set of forceps. Then, they grasped the rubber bands with the other set of forceps and placed them on the outer column. After four rubber bands were placed on the exterior columns, they returned the rubber bands to the central columns one by one. We measured the time from the start of training to return all rubber bands to the primary columns and used the performance time to score the da Vinci robot’s operating ability. This assesses the trainee’s overall ability to manipulate the instrument, coordination, and camera movement awareness.

### Training with the dVSS simulator

Fifteen students performed training using the dVSS simulator for 4 h a day for 5 days. The training started with the pick-and-place practice and then advanced to the camera-targeting practice, peg board practice, and match board practice as the level of difficulty gradually increased [13]. When the overall score exceeded 80 %, they proceeded to the next training. The overall score based on the individual trainings was automatically calculated by the simulator. A total score of 80 % or higher was considered by the simulator system to have “excellent” skills, indicating that the trainee acquired sufficient techniques from the training. Students who completed these practices were trained on more difficult tasks as time allowed.

### Subjective assessment of motion sickness

Each day before training, students assessed the degree of motion sickness using the Visual Analog Scale (VAS) and Simulator Sickness Questionnaire (SSQ). The VAS quantifies the degree of sickness experienced from 0 (no symptom) to 100 (worst) (Supplementary Fig. 1) [14]. The SSQ is a questionnaire used to evaluate motion sickness, especially when using the simulator (Supplementary Fig. 2) [15]. The SSQ was designed to assess three elements of motion sickness: nausea, oculomotor strain, and disorientation [15]. Additionally, motion sickness was assessed at the end of each day’s training using the VAS and SSQ. All scores were reported for 5 days.

### Statistical assessment

All data are shown as the mean ± standard deviation. We used chi-square and t-tests to compare background characteristics. Changes in time to complete the skill drill were analyzed using the paired t-test. Regarding VAS and SSQ scores, we used the Bonferroni adjusted paired t-test to compare motion sickness before and after training each day. In addition, repeated one-way analysis of variance was used to compare the change in motion sickness during the study period. When a statistically significant effect was found, the difference was determined using the Tukey post hoc test.

All statistical data were analyzed using the JMP 13.2 software (SAS Institute Inc., Cary, NC, USA), with *p* < 0.05 as statistically significant.

## Results

### Change in da Vinci robot skill due to the implementation of training with a VR simulator

The trained group received 20 h of practice using the VR simulator; the same training (skill drill) was performed again with the da Vinci robot, and the operation time was recorded.

There was no difference in age or video game experience between the trained and non-trained groups (Table 1). The non-trained group tended to have more male medical students than the non-trained group; however, there was no significant difference (*p* = 0.223). Before training with the VR simulator, there was no significant difference in completion time between the trained and non-trained groups (293.9 ± 72.4 and 350.5.4 ± 125.3 s, respectively; *p* = 0.142). The completion time of skill drill training was significantly shorter post-training than pre-training in the trained group (293.9 ± 72.4 versus [vs.] 143.6 ± 18.4 s; *p* < 0.001). Although the non-trained group showed a significant reduction in completion time in the latter part of the test, the difference was less than that of the training group (350.5 ± 125.3 vs. 257.1 ± 70.0 s; *p* = 0.003). In addition, the time to complete the final procedure was significantly shorter post-training in the trained group than in the non-trained group (143.6 ± 18.4 vs. 257.1 ± 70.0 s; *p* < 0.001).

### Subjective motion sickness and change caused by the VR simulator

**Fig. 2:**
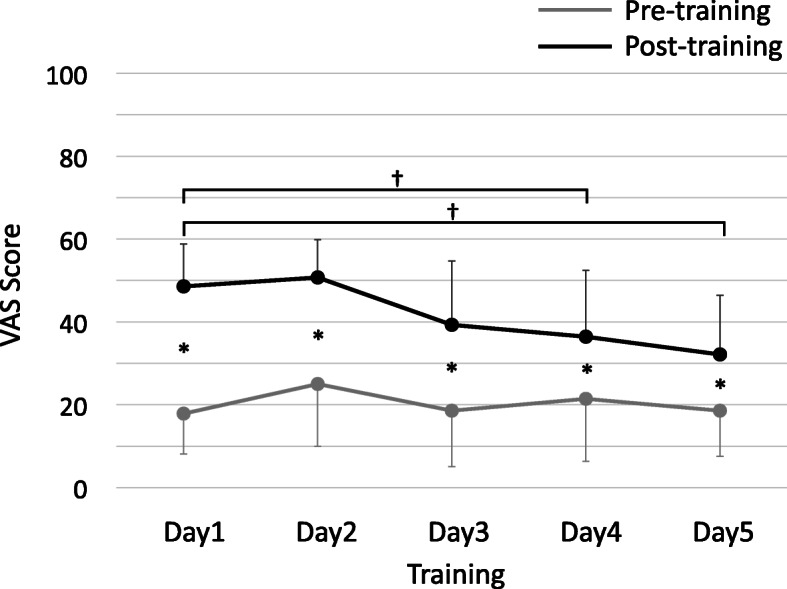
Changes in VAS scores with training. The change in VAS scores with training is shown. The vertical axis shows the VAS score, and the horizontal axis shows the training schedule. The gray and black plots show the pre-training and pre-training VAS scores (mean ± standard deviation), respectively. *A significant (*p* < 0.05) difference in scores before and after training on the same day. †A significant (*p* < 0.05) difference in scores compared to the first day. VAS, Visual Analog Scale

**Fig. 3:**
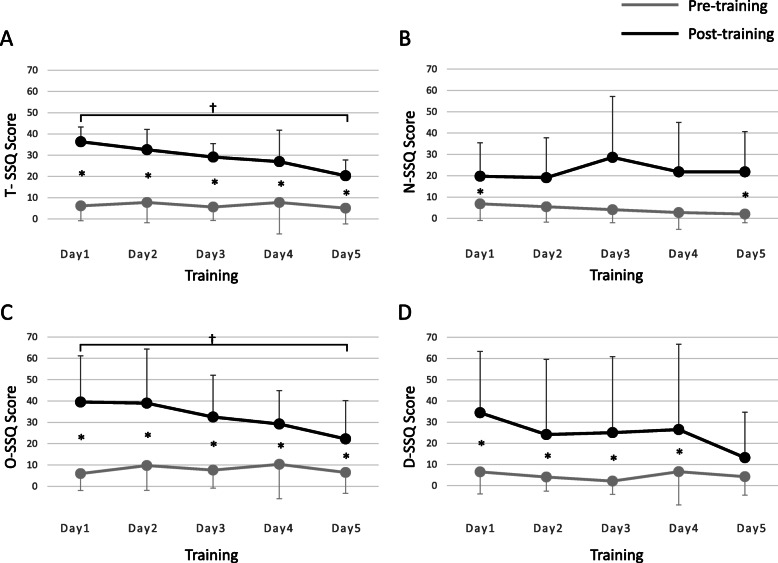
Changes in SSQ scores with training. Changes in SSQ scores with training are shown. The vertical axis shows the SSQ score, and the horizontal axis shows the training schedule. Gray and black plots show SSQ scores before and after training (mean ± standard deviation), respectively: A, total score (T-SSQ); B, nausea subscore (N-SSQ); C, oculomotor subscore (O-SSQ); and D, disorientation subscore (D-SSQ). *A significant (*p* < 0.05) difference in scores before and after training on the same day. †A significant (*p* < 0.05) difference in scores compared to the first day. SSQ, Simulator Sickness Questionnaire

The results of the 5-day training and motion sickness assessment are shown in Figs. 2 and 3, respectively. The VAS showed a significant exacerbation of sickness after the initial training, with mean values of 17.9 ± 1.0 and 48.6 ± 1.0 points before and after the training, respectively (*p* < 0.001, Fig. 2). The VAS score showed that the VR simulator training significantly exacerbated motion sickness until the final day of training.

Additionally, when we examined whether the VAS scores would change as the training progressed, there were no significant differences in the pre-training scores. However, the post-training score significantly improved with increasing frequency of training (*p* < 0.001), indicating that repeated practice improved motion sickness. Especially when compared to the first day of training, post-training motion sickness was significantly improved on the fourth and fifth days (*p* = 0.013 and *p* = 0.004, respectively).

Then, we conducted the same study using the SSQ (Fig. 3). As regards overall motion sickness (T-SSQ), we observed that motion sickness worsened significantly on the first training day and lasted considerably until the fifth day (Fig. 3 A). Moreover, motion sickness did not change between the first day and last day of pre-training (*p* = 0.906); however, fatigue post-training significantly improved with repeated practice (*p* = 0.023). Especially when compared to the first day of training, post-training motion sickness was significantly improved on the fifth day (*p* = 0.012). These results were consistent with those of the VAS scores. Furthermore, we calculated the subscores of the SSQ. Nausea (N-SSQ) was exacerbated after each training session (Fig. 3B); however, its statistical significance was weaker than that for the other subscores. There was no improvement of N-SSQ post-training even if the practice was continued (*p* = 0.921). The oculomotor subscore (O-SSQ) of post-training was as well significantly worse than that of pre-training from the first day to the final day (Fig. 3 C). The O-SSQ of post-training was significantly improved with repeated practice (*p* = 0.025), indicating that oculomotor strain improved with the training. In addition, oculomotor strain significantly improved on the fifth day of training. Finally, the disorientation subscore (D-SSQ) score showed a significant difference before and after training, except on the final day (Fig. 3D). Moreover, there was no improvement in the D-SSQ post-training even with repeated training (*p* = 0.150). Thus, the simulator training had no significant effect on nausea- and disorientation-related motion sickness.

## Discussion

The advantages of RAS include a tenfold magnified 3D field of view, which allows detailed organ observation and sensitive surgical manipulation as the extremely flexible forceps can reflect the surgeon’s movements in real time [16, 17]. Additionally, robust image stabilization can inhibit the inadvertent movement of the forceps, making it possible to operate safely, even for elderly surgeons. However, surgical robots have an extremely different operating system than conventional laparoscopic surgery instruments, and without adequate training in their operation, dangerous operations can lead to severe complications [2]. Particularly, since the da Vinci robot does not have tactile feedback, the possibility of severe tissue damage cannot be ruled out if the forceps are moved outside the field of view [18]. Therefore, it is strongly recommended that sufficient time for adequate training is given before the start of surgery to ensure that safe surgical procedures are performed [4].

Since the da Vinci robot produces a 3D image, there is the potential for unusual motion sickness fatigue when the surgeon operates the system. Motion sickness may occur during VR simulator training and during actual surgical operations, and surgeons sometimes complain after using the robot. The mechanism of the onset of motion sickness is not well understood; however, the theory of sensory discrepancy is considered as the main one [19]. It is hypothesized that a disparity between the vestibular, visual, and somatosensory senses’ experience and the actual senses confuses spatial perception, excites the sympathetic nervous system, and causes motion sickness symptoms. In particular, viewing 3D images using binocular stereopsis often causes unpleasant symptoms, such as headache, vomiting, and eyestrain, depending on the viewing conditions. Further, ataxia has been reported in the case of motion sickness due to VR simulators [20]. Humans perceive 3D images by adjusting radiation and the crystalline lens simultaneously. The 3D images are composed of incredible pictures in which the gaze is fixed, ignoring the output adjustment. There is no report on what kind of motion sickness is induced by the da Vinci robot operation with 3D images.

Our study presents several notable findings. First, we observed a significant improvement in the manipulation time for the students who were trained with a VR simulator. Therefore, the VR simulator was useful for improving the RAS technique even for students without surgical experience. Second, we found that motion sickness worsened after the training according to the VAS and T-SSQ scores. In contrast, continuous training with the VR simulator reduced motion sickness caused by 3D manipulation. In summary, practice with the VR simulator could improve not only the technique of the operation but also the student’s fatigue because of the process of the da Vinci robot.

When we evaluated the subscores of the SSQ, training with a VR simulator exacerbated nausea (N-SSQ), oculomotor (O-SSQ), and disorientation (D-SSQ). Furthermore, the O-SSQ post-training improved significantly with repeated practice. Conversely, the N-SSQ and D-SSQ post-training did not improve after a short training period. Therefore, oculomotor strain improved with repeated training; however, nausea and disorientation did not improve with repeated practice. In addition, the N-SSQ score showed little difference before and after training on the same day; therefore, training with the VR simulator may not induce nausea.

With the increase in the number of RASs in recent years, students have more opportunities to be exposed to RAS [21, 22]. The benefits of exposing medical students to RAS and the simulators are substantial [23]. Medical students can fully learn the latest technologies that are becoming mainstream in surgery, and they will be able to provide more appropriate information to their patients when they become doctors. Moreover, it can lead to increased motivation for the new generation of surgeons. Therefore, we believe that training medical students with VR simulators is significant for medical education. In fact, the medical students who participated in this program stated that they would like to become experts in RAS in the future.

This study had a few limitations. First, the number of subjects was small; thus, further investigation with a larger sample size is required. However, education programs were limited by the COVID-19 pandemic; hence, we could not accumulate enough samples. Second, we conducted a study of students with no prior surgical experience. Further research is needed to determine whether motion sickness is similar between inexperienced and experienced surgeons. Additionally, this training session lasted only 5 days, and it would be desirable to investigate schedules further. Despite these limitations, the significant improvement in motion sickness after using the VR simulator repeatedly showed its usefulness in terms of reducing motion sickness.

## Conclusions

In conclusion, we showed that training with the VR simulator for the da Vinci robot induced motion sickness, and we demonstrated that repeated training using the VR simulator improved motion sickness. Training using the VR simulator not only develops the operator’s RAS technique but also potentially improves the operator’s motion sickness.

## Supplementary information

Additional file 1 Figure S1

Additional file 2 Figure S2

## Data Availability

The datasets used and/or analyzed during the current study are available from the corresponding author on reasonable request.

## Abbreviations

3D: three-dimensional
COVID-19: coronavirus disease 2019
D-SSQ: disorientation subscore of Simulator Sickness Questionnaire
dVSS: da Vinci Skills Simulator
N-SSQ: nausea subscore of Simulator Sickness Questionnaire
O-SSQ: oculomotor subscore of Simulator Sickness Questionnaire
RAS: robotic-assisted surgery
SSQ: Simulator Sickness Questionnaire
T-SSQ: overall motion sickness of Simulator Sickness Questionnaire
VAS: Visual Analog Scale
VR: virtual reality
vs.: versus
