# Supplementary material for: Improvement of three-dimensional motion sickness using a virtual reality simulator for robot-assisted surgery in undergraduate medical students: A prospective observational study
Source: BMC Med Educ. 2021 Sep 21;21:498. doi: 10.1186/s12909-021-02872-9 (PMC8454008; doi:10.1186/s12909-021-02872-9)
Supplement: Supplementary file 1 — Figure S1 [file 12909_2021_2872_MOESM1_ESM.pptx]

## Slide 1
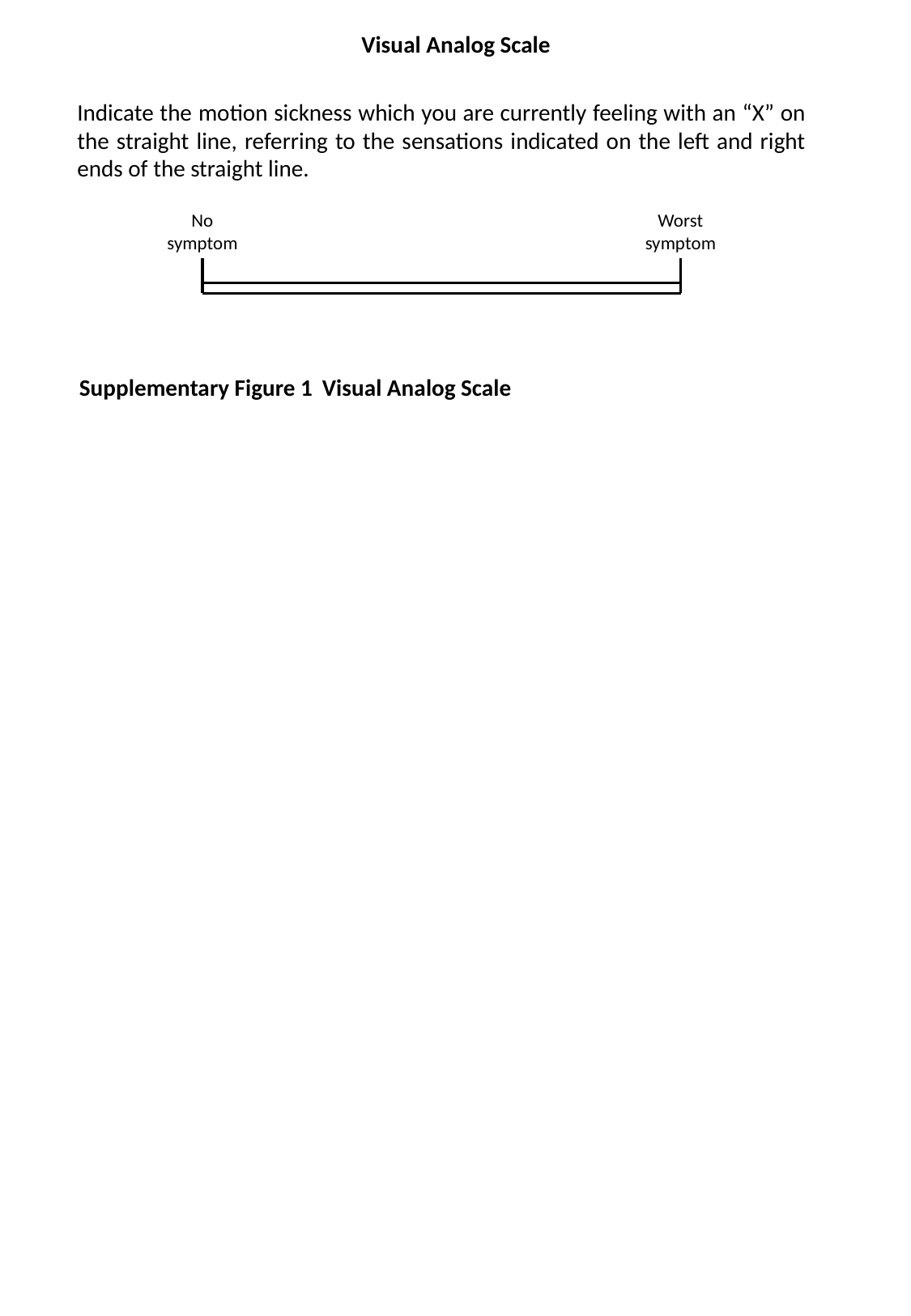

Visual Analog Scale
Indicate the motion sickness which you are currently feeling with an “X” on the straight line, referring to the sensations indicated on the left and right ends of the straight line.
No
symptom
Worst symptom
Supplementary Figure 1	Visual Analog Scale
