# Supplementary material for: Improvement of three-dimensional motion sickness using a virtual reality simulator for robot-assisted surgery in undergraduate medical students: A prospective observational study
Source: BMC Med Educ. 2021 Sep 21;21:498. doi: 10.1186/s12909-021-02872-9 (PMC8454008; doi:10.1186/s12909-021-02872-9)
Supplement: Supplementary file 2 — Figure S2 [file 12909_2021_2872_MOESM2_ESM.pptx]

## Slide 1
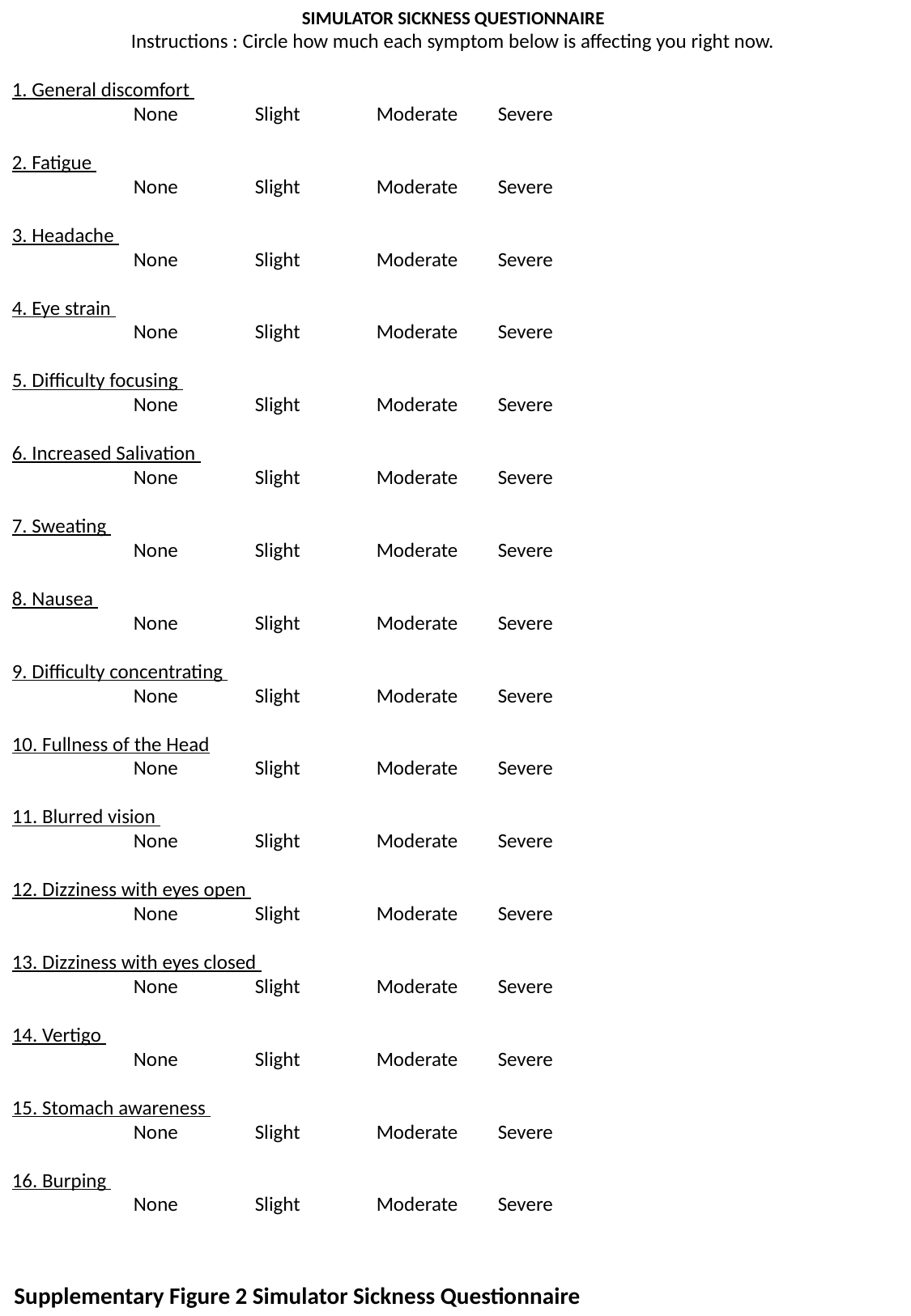

SIMULATOR SICKNESS QUESTIONNAIRE
Instructions : Circle how much each symptom below is affecting you right now.
1. General discomfort
	None 	Slight 	Moderate 	Severe
2. Fatigue
	None 	Slight 	Moderate 	Severe
3. Headache
	None 	Slight 	Moderate 	Severe
4. Eye strain
	None 	Slight 	Moderate 	Severe
5. Difficulty focusing
	None 	Slight 	Moderate 	Severe
6. Increased Salivation
	None 	Slight 	Moderate 	Severe
7. Sweating
	None 	Slight 	Moderate 	Severe
8. Nausea
	None 	Slight 	Moderate 	Severe
9. Difficulty concentrating
	None 	Slight 	Moderate 	Severe
10. Fullness of the Head
	None 	Slight 	Moderate 	Severe
11. Blurred vision
	None 	Slight 	Moderate 	Severe
12. Dizziness with eyes open
	None 	Slight 	Moderate 	Severe
13. Dizziness with eyes closed
	None 	Slight 	Moderate 	Severe
14. Vertigo
	None 	Slight 	Moderate 	Severe
15. Stomach awareness
	None 	Slight 	Moderate 	Severe
16. Burping
	None 	Slight 	Moderate 	Severe
Supplementary Figure 2 Simulator Sickness Questionnaire
